# The Role of *Helicobacter pylori* Heat Shock Proteins in Gastric Diseases’ Pathogenesis

**DOI:** 10.3390/ijms26115065

**Published:** 2025-05-24

**Authors:** Olga Maria Manna, Celeste Caruso Bavisotto, Melania Ionelia Gratie, Provvidenza Damiani, Giovanni Tomasello, Francesco Cappello

**Affiliations:** 1 Pathologic Anatomy Unit, Department of Health Promotion, Mother and Child Care, Internal Medicine and Medical Specialties, University of Palermo, 90127 Palermo, Italy; olgamaria.manna@unipa.it; 2 Department of Biomedicine, Neurosciences and Advanced Diagnostics (BIND), Institute of Human Anatomy and Histology, University of Palermo, 90127 Palermo, Italy; celeste.carusobavisotto@unipa.it (C.C.B.); melania.gratie@gmail.com (M.I.G.); 3 Risk Management and Quality Unit, Hospital University “Paolo Giaccone”, 90127 Palermo, Italy; donatelladamiani@alice.it (P.D.); giovanni.tomasello@unipa.it (G.T.)

**Keywords:** gastric mucosa, muco-microbiotic layer, *Helicobacter pylori*, heat shock proteins, antibiotic resistance, microbiota

## Abstract

*Helicobacter pylori* (*H. pylori*) is a Gram-negative bacterium that colonizes the human stomach and is associated with several gastric diseases, including gastritis, peptic ulcer disease, and gastric cancer. The bacterium’s ability to thrive in the harsh gastric environment is due, to some extent, to its stress response mechanisms, with its heat shock proteins (HSPs) playing a putative, yet not fully understood, role in these adaptive processes. HSPs are a family of molecules, highly conserved throughout phylogenesis, that assist in protein folding, prevent aggregation, and ensure cellular homeostasis under stressful conditions. In *H. pylori*, HSPs contribute to survival in the stomach’s acidic environment and oxidative stress. Furthermore, they aid in the bacterium’s ability to adhere to gastric epithelial cells, modulate the host immune response, and form biofilms, all contributing to chronic infection and pathogenicity. The role of microbial HSPs in antibiotic resistance has also emerged as a critical area of research, as these proteins help stabilize efflux pumps, protect essential proteins targeted by antibiotics, and promote biofilm formation, thereby reducing the efficacy of antimicrobial treatments. Among bacterial HSPs, GroEL and DnaK are probably the major proteins that control most of the *H. pylori*’s functioning. Indeed, both proteins possess remarkable acid resistance, high substrate affinity, and dual roles in protein homeostasis and host interaction. These features make them critical for *H. pylori*’s adaptation, persistence, and pathogenicity in the gastric niche. In addition, recent findings have also highlighted the involvement of HSPs in the crosstalk between *H. pylori* and gastric epithelial cells mediated by the release of bacterial outer membrane vesicles and host-derived exosomes, both of these extracellular vesicles being part of the muco-microbiotic layer of the stomach and influencing cellular signalling and immune modulation. Considering their critical role in the survival and persistence of bacteria, microbial HSPs also represent potential therapeutic targets. Strategies aimed at inhibiting microbial HSP function, combined with conventional antibiotics or developing vaccines targeting microbial HSPs, could provide new avenues for the treatment of *H. pylori* infections and combat antibiotic resistance. This review explores the multifaceted roles of microbial HSPs in the pathogenesis of *H. pylori*, highlighting their contributions to bacterial adhesion, immune evasion, stress response, and antibiotic resistance.

## 1. Introduction

*Helicobacter pylori* (*H. pylori*) is a Gram-negative, spiral-shaped bacterium that colonizes the human stomach and is associated with various gastric disorders, including gastritis, peptic ulcer disease, and gastric cancer [1]. To survive in the harsh gastric environment, *H. pylori* employs several adaptive mechanisms, including the production of its heat shock proteins (HSPs), which assist the bacterium in protein folding, prevent aggregation, and helping bacteria withstand environmental stresses [2]. HSPs are a family of conserved proteins found in nearly all living organisms, including bacteria, archaea, and eukaryotes [3]. They play a critical role in protecting cells from stress-induced damage by ensuring proper protein folding and preventing the accumulation of misfolded or aggregated proteins [4]. In pathogenic bacteria such as *H. pylori*, HSPs contribute to survival under hostile conditions and to the bacterium’s ability to colonize and persist in the gastric mucosa. Their involvement in bacterial stress responses, immune modulation, and biofilm formation further highlights their importance in pathogenesis [5,6]. Given the growing concern about antibiotic resistance in *H. pylori*, understanding the role of HSPs in bacterial adaptation and persistence can be useful for developing novel therapeutic strategies. This review aims to explore the role of *H. pylori* HSPs in human gastric pathogenesis, focusing on adhesion, immune evasion, stress response, and antibiotic resistance. We also highlight the role of the crosstalk between bacterial and human extracellular vesicles containing HSPs in the homeostasis of gastric mucosa and its alteration. A deeper understanding of these mechanisms may provide novel insights into potential therapeutic interventions targeting *H. pylori* infections.

## 2. The Evolution of Heat Shock Proteins: From Very Ancestral Forms of Life to Humans

HSPs (most of which are molecular chaperones) are a highly conserved group of molecules essential for protein homeostasis in all domains of life. Although they were first discovered serendipitously by Ritossa due to their increased expression during heat stress and their role in protecting cellular proteins [7], further studies have clarified that their primary functions include the facilitation of proper protein folding, prevention of protein aggregation, and assistance in protein refolding or degradation under stress conditions [8]. The evolutionary history of HSPs is deeply intertwined with the emergence and diversification of life, from primordial microbial life forms inhabiting extreme environments to complex multicellular organisms, culminating in their multifaceted roles in human physiology and pathology.

Indeed, the origin of HSPs likely dates back to the earliest stages of life on Earth, over 3 billion years ago [9,10]. Primitive life forms, particularly thermophilic archaea and bacteria, evolved in high-temperature environments. These extreme conditions posed a constant threat to protein stability, exerting selective pressure for the evolution of mechanisms that could maintain proteome integrity [11]. Small HSPs, Hsp60 (chaperonins), and Hsp70 families are believed to be among the most ancient, with homologs identified in all domains of life. Their robust thermostability and ATP-independent or ATP-dependent mechanisms for protein stabilization made them indispensable for early cellular survival.

In prokaryotes, HSPs such as GroEL (member of the Hsp60 family), DnaK (member of the Hsp70 family), and ClpB (member of the Hsp100 family) are organized into stress response systems that are tightly regulated at the transcriptional level, often under the control of heat shock sigma factors (e.g., σ^32^ in *Escherichia coli*) [12,13]. The conservation of structural motifs and chaperone function suggests that the basic mechanisms of protein quality control were established early and maintained due to their fundamental importance.

The transition from unicellular to multicellular life introduced novel challenges, including tissue specialization, longer protein lifespans, and increased proteomic complexity. In eukaryotes, HSPs diversified not only in sequence and cellular localization but also in functional specialization. The evolution of compartmentalized cells in early eukaryotes necessitated the targeting of HSPs to organelles such as mitochondria, the endoplasmic reticulum, and the nucleus. For example, mitochondrial Hsp60 plays a critical role in the folding of proteins imported into the organelle, reflecting both an evolutionary adaptation and a remnant of the bacterial endosymbiotic origin of mitochondria [14].

In metazoans, the HSP gene families expanded further, with the emergence of new members and isoforms, some of which acquired roles beyond classical chaperoning. These include participation in developmental processes, apoptosis regulation, and other homeostatic processes, including crosstalk among cells [15,16]. Hsp90, for instance, became a central hub in the regulation of signal transduction pathways, interacting with kinases, hormone receptors, and transcription factors in a highly ATP-dependent manner. Its involvement in buffering phenotypic variation has led to the hypothesis that Hsp90 acts as a “capacitor of evolution” [17].

In mammals, and particularly in humans, HSPs are involved in both physiological and pathological processes. They contribute to the maintenance of proteostasis under stress (e.g., fever, hypoxia, oxidative stress), and are upregulated in response to inflammation and infection [18]. Beyond their intracellular functions, several HSPs are found extracellularly, including those released from cells by alternative pathways (i.e., extracellular vesicles) [19], acting as danger-associated molecular patterns (DAMPs) that activate the innate immune system. Hsp60 and Hsp70, in particular, have been implicated in modulating immune responses during infection and inflammation as well as in autoimmune diseases [20,21,22]. Moreover, the expression of HSPs in cancer cells is often dysregulated, contributing to tumour survival, metastasis, and resistance to therapy, some of these diseases framed nosologically as chaperonopathies [21,22]. We further discuss about that in this review.

## 3. Bacterial Heat Shock Proteins

Bacterial HSPs are fundamental components of the cellular machinery that safeguard proteostasis under both normal and stress-induced conditions. These molecular chaperones, including representatives of the Hsp100 (ClpB), Hsp70 (DnaK), Hsp60 (GroEL), Hsp40 (DnaJ), and small HSP families, facilitate the correct folding of nascent and stress-denatured proteins, prevent irreversible aggregation, and aid in protein refolding or degradation [23].

Among them, the DnaK–DnaJ–GrpE system (equivalent to the Hsp70–Hsp40 complex in eukaryotes) forms a highly conserved and ATP-dependent triad responsible for the dynamic recognition and release of unfolded proteins. DnaK is also involved in the autoregulatory feedback of the heat shock response through its interaction with σ^32^ (RpoH), the heat shock sigma factor that governs the transcription of major HSP genes in bacteria [24].

Similarly, the GroEL–GroES system (equivalent to Hsp60–Hsp10 complex in eukaryotes) is a hallmark of bacterial chaperonin function. GroEL forms a barrel-shaped tetradecamer that encapsulates non-native polypeptides, providing a secluded environment for ATP-driven folding, while the co-chaperonin GroES caps the chamber to complete the folding cycle [25]. These chaperonins are indispensable for bacterial viability, particularly under conditions that denature cellular proteins such as heat shock, oxidative stress, or pH shifts.

Small HSPs, although ATP-independent, also play important roles in early stress responses by binding to unfolding proteins and preventing their aggregation until ATP-dependent systems can act.

The regulation of the bacterial heat shock response is tightly controlled at the transcriptional level, predominantly through σ^32^, whose expression and stability are modulated by cellular stress levels and chaperone availability [26]. Under non-stressed conditions, σ^32^ is rapidly degraded by the FtsH protease in a DnaK–GroEL-dependent manner; however, under stress, these chaperones are titrated away by misfolded proteins, allowing σ^32^ to accumulate and activate the transcription of heat shock genes.

Beyond their canonical roles as chaperones, bacterial HSPs have emerged as multifunctional proteins involved in diverse physiological and pathological processes. In several pathogenic bacteria, HSPs such as GroEL and DnaK are found on the cell surface or secreted extracellularly, where they function as virulence factors [27]. These “moonlighting” functions include binding to host extracellular matrix proteins, promoting adhesion and invasion, and modulating the host immune response.

For instance, surface-localized GroEL has been shown to stimulate pro-inflammatory cytokine release and contribute to immune evasion, while DnaK interacts with host proteins involved in antigen presentation and inflammation [27]. Such findings have sparked interest in bacterial HSPs as potential targets for antimicrobial agents or vaccine development [28]. Their high degree of conservation and immunogenicity make them attractive candidates, although their structural similarity to human HSPs poses challenges in specificity and potential cross-reactivity.

In addition, since HSPs are essential molecules that help bacteria survive environmental stresses (e.g., acid, heat, oxidative conditions), without them, proteins misfold and bacterial cells die. Biofilm formation itself is a stress-response strategy, but it requires functional HSPs to fold and maintain the enzymes and matrix components essential for biofilm development [29]. Thus, the loss of HSPs compromises both immediate stress resistance and the ability to form protective biofilms. This is particularly true for HP, as better described below.

Overall, bacterial HSPs represent a remarkable example of molecular adaptation and functional diversification. Their evolutionarily conserved roles in protein folding are matched by an expanding repertoire of regulatory and virulence-associated functions, underscoring their central importance not only in bacterial cell biology but also in host–pathogen interactions.

## 4. Heat Shock Proteins in *H. pylori*

The *H. pylori* genome contains genes encoding HSP homologs such as GroEL, GroES, DnaK, DnaJ, GrpE, HtpG, and CbpA [30,31]. These proteins are also essential for bacterial survival and pathogenicity, playing key roles in cellular homeostasis, stress response, and host interactions. Each of these HSP also contributes to distinct aspects of bacterial physiology, and their coordinated functions enable *H. pylori* to thrive under extreme conditions.

In general terms, the timeline for *H. pylori* infection involves multiple stages, including colonization, adaptation, and establishment within the gastric mucosa. While specific data on the exact duration for HSPs to facilitate infection are limited, studies indicate that *H. pylori* can induce host cellular responses within hours of contact. For instance, in monocyte cultures infected with H. pylori, nuclear accumulation of heat shock factor 1 was observed at 6 h post-infection, with significant binding to the HSP70/DnaK gene promoter occurring at 24 h [32]. This suggests that *H. pylori*’s interaction with host cells and the subsequent stress responses, including HSP involvement, commence relatively quickly after infection.

Moreover, *H. pylori* upregulates its own HSPs in response to various environmental stressors encountered within the gastric environment. In a few words, key conditions include the following: (1) heat shock (exposure to elevated temperatures, such as a shift from 37 °C to 42 °C, leads to the upregulation of several HSP genes, including GroEL, GroES, DnaK, and CbpA); (2) acidic pH (the acidic conditions of the stomach stimulate *H. pylori* to increase HSPs’ expression to maintain protein stability and function); (3) oxidative stress (reactive oxygen species, produced during the host immune response, induce HSPs’ expression in *H. pylori* as a protective mechanism) [33]. These stress-induced expressions of HSPs enable *H. pylori* to adapt and survive in the hostile gastric environment, facilitating persistent infection.

Table 1 summarises how human HSPs differ from H. pylori’s HSPs in several key aspects, despite sharing conserved structural motifs and functional similarities as molecular chaperones. Table 2 summarises the principal functions of the main HSPs in *H. pylori*’s physiology and pathophysiology.

For example, GroEL and GroES function as molecular chaperones facilitating protein folding and refolding, particularly under stress conditions [34]. GroEL also interacts with host cells in adhesion and immune modulation [35]. Beyond its role in protein stability, GroEL has been implicated in virulence due to its ability to stimulate host immune responses and contribute to chronic inflammation [36]. Also, DnaK assists in protein folding, stabilization, and refolding of denatured proteins. It regulates stress responses and enhances bacterial survival [37]. Additionally, it plays a role in modulating enzymatic activities essential for bacterial metabolism [38].

**Table 1:** This table summarises the main differences between human and *H. pylori*’s HSPs referring to some aspects further discussed in this review.

Key Aspects	Human HSPs	*H. pylori* HSP	References
**Origin and evolution**	Human HSPs are eukaryotic proteins, encoded by the human genome, and have evolved within the context of complex, compartmentalized cells and multicellular organisms.	*H. pylori* HSPs are prokaryotic, adapted to the bacterium’s specific intracellular and extracellular environments, including the highly acidic gastric niche.	[18,19,30,31]
**Sequence homology with divergence**	Despite structural conservation (e.g., GroEL–DnaK in *H. pylori* vs. Hsp60–Hsp70 in humans), there are significant sequence differences that distinguish the bacterial from the human homologs. These differences are critical for pathogen-specific targeting by the immune system and for the development of therapeutic interventions.		[39]
**Function and cellular context**	While both human and bacterial HSPs assist in protein folding, stress response, and prevention of protein aggregation, human HSPs also have broader roles in apoptosis and cancer biology.	In contrast, *H. pylori* HSPs contribute to bacterial survival under stress, colonization, immune evasion, and biofilm formation.	[34,35,37,38]

**Table 2:** This table summarises the main HSP family members in *H. pylori*, their primary functions, and their principal roles in the pathogenesis of the disease. Concerning their roles in pathogenesis, it is further explained in Section 5 of this review as well as in the Table in Section 5.3.

HSP Family	Bacterial Protein	Main Function	Main Role in *H. pylori* Pathogenesis	References
** Hsp10 **	GroES	Regulates urease activity and epithelial cell adhesion	Adhesion of *H. pylori* to primary human gastric epithelial cells facilitates colonization of stomach	[30]
** Hsp60 **	GroEL	Facilitates protein folding and refolding of denatured proteins	Involved in host cell adhesion and immune modulation, stimulates inflammatory responses, and contributes to virulence	[35,36]
** Hsp70 **	DnaK	Assists in protein folding, stabilizes denatured proteins, and regulates stress responses	Supports bacterial survival under stress, regulates enzymatic activity, and modulates metabolic mechanisms	[37,38]

## 5. Role of Heat Shock Proteins in *H. pylori* Pathogenesis

### 5.1. Adhesion and Colonization

Adherence to gastric epithelial cells is a crucial step in *H. pylori* colonization. GroEL has been implicated in bacterial adhesion by interacting with host cell surface molecules such as integrins [40]. This interaction facilitates bacterial attachment and colonization, allowing *H. pylori* to establish a persistent infection. The ability of *H. pylori* to form a stable attachment to gastric epithelial cells ensures its long-term survival despite the continuous turnover of gastric mucosal cells and exposure to host immune defences.

Moreover, HSPs may contribute to biofilm formation, a key factor in bacterial persistence and antibiotic resistance [41], primarily through their role as molecular chaperones, ensuring the correct folding, stabilization, and functionality of proteins that are essential for biofilm development. While HSPs are not classical transcriptional regulators, their influence on biofilm formation is multifactorial, throughout the stabilization of matrix-associated proteins [42], modulation of stress-response pathways [43] and indirect effects on gene regulation and signalling pathways [44].

Biofilms provide a protective niche for bacteria, shielding them from host immune responses and antimicrobial agents [45,46,47]. Studies have shown that GroEL and other chaperone proteins enhance biofilm formation, promoting *H. pylori* survival in the gastric environment [48,49,50]. Biofilm homeostasis is crucial in *H. pylori* infections, contributing to decreased antibiotic efficacy [51,52] and, putatively, to recurrent infections, although this link has not been clearly established in the context of H. pylori. Finally, HSPs may influence the regulation of biofilm-related genes in *H. pylori*, further highlighting their role in bacterial persistence [53].

### 5.2. Resistance to Environmental Stress

*H. pylori* face extreme conditions in the stomach, including high acidity, oxidative stress, and nutrient scarcity. Its HSPs play a crucial role in protecting bacterial cells from these challenges. The acidic environment of the stomach poses a significant challenge for *H. pylori*. GroEL and DnaK contribute to acid resistance by maintaining protein stability and refolding denatured proteins [54]. Additionally, they may interact with urease, an enzyme that neutralizes gastric acid by producing ammonia, thereby enhancing bacterial survival.

During infection, *H. pylori* is exposed to reactive oxygen species (ROS) produced by the host immune system. HSPs may mitigate oxidative damage by stabilizing proteins, enhancing antioxidant enzyme activity, and reducing protein aggregation [55]. The ability to counteract oxidative stress is essential for bacterial survival, as host immune cells generate ROS as a defence mechanism against pathogens. By repairing oxidatively damaged proteins and maintaining redox balance, HSPs help *H. pylori* evade host defences and persist within the gastric mucosa [56,57,58].

Although *H. pylori* resides in a relatively stable temperature environment, putative fluctuations in temperature (such as those that may occur during a fever or, specifically for the stomach, for the introduction of hot or cold food) could still pose a stress challenge. HSPs can assist in maintaining protein homeostasis under varying stress conditions—such as chemical (i.e., hyperacidity) and physical (e.g., shear stress exerted by gastric content flow) stressors—ensuring that critical bacterial functions remain intact [59]. These stress response mechanisms allow *H. pylori* to survive and proliferate despite the hostile gastric environment.

### 5.3. Modulation of the Immune Response

HSPs support *H. pylori* survival and influence on the host immune response. In particular, GroEL has been shown to interact with Toll-like receptors (TLRs) on host cells, triggering immune responses that contribute to chronic inflammation [60]. GroEL activates TLR2 and TLR4, producing pro-inflammatory cytokines such as IL-8 [61]. Chronic exposure to these cytokines results in persistent inflammation, a hallmark of *H. pylori*-induced gastritis and peptic ulcer disease.

However, we should be aware that *H. pylori* HSPs share structural similarities with human HSPs, potentially leading to autoimmune responses [62,63,64]. These molecular mimicry phenomena, particularly for GroEL and GroES, have been implicated in the pathogenesis of gastric disorders and systemic autoimmune diseases associated with *H. pylori* infection [65]. Finally, HSPs may assist *H. pylori* in evading host immune responses by modulating apoptosis and inhibiting macrophage function, thereby promoting bacterial persistence. This and other information are summarised in Table 3.

## 6. HSP-Mediated Crosstalk Between *H. pylori* and Gastric Mucosa: The Role of Extracellular Vesicles

Analogously to the bowel, in the stomach, we can also describe a muco-microbiotic layer as the complex interface between the host gastric mucosa and the luminal environment [66,67]. In detail, it consists of a dynamic barrier formed by the mucous secreted by surface mucous epithelial cells and colonized by a specialized microbial community adapted to the acidic gastric niche, rather than of extracellular vesicles produced by host cells/microorganisms [68]. Hence, this layer not only protects the mucosa from physical and chemical insults but also plays an active role in modulating immune responses and maintaining mucosal homeostasis. *H. pylori*, one of the putative dominant microorganisms in this environment during its infection, can remodel the muco-microbiotic layer by altering its microbiota composition and in turn influencing gastric physiology and pathophysiology [1]. Understanding better this micro-ecosystem may open new perspectives in research and practice.

Therefore, the gastric mucosa represents a dynamic interface where host and microbial factors engage in a complex molecular dialogue. As we discuss in this review, among the key players in this interaction are HSPs, such as Hsp60, Hsp70, and Hsp90, which serve not only as intracellular chaperones but also as extracellular signalling molecules [69]. In the context of *H. pylori* infection, both bacterial and host-derived HSPs, such as GroEL/Hsp60 and/or DnaK/Hsp70, can be released into the extracellular space via membrane-bound vesicles: like other bacteria *H. pylori* produces outer membrane vesicles (OMVs), while gastric epithelial cells secrete exosomes as well as other types of extracellular vesicles [70]. The crosstalk between these vesicle-associated HSPs is emerging but not yet fully understood as a component of the pathophysiological processes in the gastric microenvironment, and this is why we would like to stress this phenomenon that, in our opinion, could contribute to immune modulation, chronic inflammation, and potentially to carcinogenesis.

OMVs are spherical proteoliposomes naturally released by Gram-negative bacteria, including *H. pylori*. These vesicles, typically ranging from 20 to 300 nm in diameter, encapsulate a rich cargo of bacterial components such as outer membrane proteins, lipopolysaccharide, enzymes, toxins, and chaperones, including GroEL and DnaK [70]. Notably, *H. pylori*-derived OMVs are enriched in virulence factors like VacA and CagA and HSPs which have been shown to maintain their immunogenic and signalling capacity even in the extracellular vesicle-associated form [71].

On the host side, gastric epithelial cells may respond to bacterial stimuli by secreting exosomes loaded with proteins, lipids, and RNA species, including host-derived HSPs such as Hsp60 [72]. These exosomes can mediate intercellular communication within the muco-microbiotic layer, as well as between epithelial cells and immune effectors localized in the lamina propria of the gastric mucosa. The bidirectional exchange of HSPs between bacterial OMVs and host exosomes could create a vesicle-based signalling network that sustains and shapes the immune microenvironment during *H. pylori* infection. Although human HSPs cannot directly counteract the pathogen, they rather aim to protect host cells and maintain homeostasis, while the presence of bacterial HSPs can trigger a hostile immune response that also involves the host’s own HSPs, creating a vicious cycle of chronic inflammation.

One of the critical aspects of this crosstalk can be the functional mimicry and immune modulation driven by HSPs from both sources. OMVs can bind to pattern recognition receptors such as TLR2 and TLR4 on gastrointestinal epithelial and immune cells [73]. This engagement leads to the activation of NF-κB and AP-1 pathways, resulting in the transcription of pro-inflammatory cytokines like IL-8 and TNF-α. However, it is not clear if these OMVs could also transfer bacterial HSPs to host cells, although this is very likely. Interestingly, host-derived exosomal Hsp60 can trigger similar signalling cascades, indicating a putative convergence of bacterial and host-derived HSP signals on shared intracellular pathways [74,75]. This convergence could amplify the inflammatory response or, paradoxically, lead to immune tolerance depending on the balance of signals received.

In addition, vesicle-mediated delivery may enhance the stability and bioavailability of HSPs in the harsh gastric environment. OMVs can protect bacterial HSPs from proteolytic degradation and acidic denaturation, allowing sustained interaction with host cells [76]. Similarly, exosomes stabilize their HSP cargo and direct it to specific recipient cells, including antigen-presenting cells in the gastric lamina propria [77]. This targeted delivery system should ensure that both bacterial and host HSPs would be not merely passive markers of stress or infection but active modulators of cell signalling, inflammation, and immune polarization.

Recent studies lead to hypothesize that gastric epithelial cells, upon exposure to GroEL-containing OMVs, may respond by upregulating their own HSP expression and increasing the release of exosomal HSPs [78]. This feedback loop might serve to protect epithelial cells from OMV-induced cytotoxicity, but at the same time, it might contribute to a pro-inflammatory microenvironment. For instance, exosomal Hsp70 has been shown to stimulate natural killer cells and macrophages via TLR2, enhancing the production of IFN-γ IL-6 and other interleukins [79,80]. Thus, the combined action of OMV- and exosome-associated HSPs could orchestrate a sustained inflammatory state, which is a hallmark of chronic *H. pylori* infection and in turn can lead to severe complications such as gastric ulcer and cancer.

A further layer of complexity arises from the potential for direct interaction or competition between bacterial OMVs and host exosomes in the muco-microbiotic layer. Both vesicle types can be internalized by epithelial cells through clathrin- or caveolin-mediated endocytosis, and they may compete for binding to the same surface receptors [81]. This competition may influence the net cellular response to infection, either tipping the balance toward inflammation or tolerance. Moreover, some data suggest that OMVs and exosomes may share common molecular signatures, such as phosphatidylserine exposure or HSP surface display [70], enabling them to hijack each other’s trafficking pathways and evade immune detection.

The concept of HSP-based crosstalk via vesicles also holds promise for therapeutic innovation. Engineering synthetic vesicles loaded with modified HSPs or HSP inhibitors should be tested as novel therapeutic strategies to modulate immune responses or restore epithelial homeostasis in chronic *H. pylori*-associated gastritis.

The interaction between *H. pylori*-derived outer membrane vesicles and host-derived exosomes may represent a finely tuned system of vesicle-mediated communication in the gastric mucosa. HSPs carried by these vesicles play central roles in mediating this crosstalk, influencing host immunity, epithelial stress responses, and disease progression. A deeper understanding of the molecular mechanisms governing this vesicular dialogue could reveal new targets for intervention in *H. pylori*-related disorders and prevent its worse complications, including gastric ulcer and cancer, as further discussed in the next sections.

## 7. HSPs and Antibiotic Resistance

Some studies permit to hypothesize that some HSPs may also play a role in the development and maintenance of antibiotic resistance in *H. pylori*. Indeed, these proteins, by contributing to bacterial stress adaptation, may enhance survival under antibiotic pressure [82]. Specifically, GroEL and DNAk have been linked to stabilizing bacterial efflux pumps, which expel antibiotics from bacterial cells, reducing their effectiveness [82,83]. Efflux pumps are essential mechanisms that allow bacteria to resist the lethal effects of antimicrobial agents by actively transporting them out of the cell before they can exert their toxic effects. The upregulation of these pumps, putatively mediated by these HSPs, may provide *H. pylori* with a robust defence against commonly used antibiotics, including amoxicillin, clarithromycin, and metronidazole, often used for treatment without success [84].

Additionally, HSPs prevent the denaturation and degradation of essential proteins, such as the ribosomal ones, that antibiotics target, thereby ensuring the continued functionality of key cellular components even in antimicrobial agents [82,85,86]. This stabilization allows *H. pylori* to maintain its structural and functional integrity, even under conditions usually lethal in other bacteria. By stabilizing essential enzymes such as urease [30,54], which plays a crucial role in neutralizing gastric acidity, HSPs may help *H. pylori* maintain its viability in the stomach environment, where antibiotics are often administered.

As previously stated, HSPs also contribute to biofilm formation, a physical barrier preventing antibiotics from reaching bacterial cells [48]. Biofilms are dense clusters of bacteria encased in a self-produced extracellular matrix, providing protection from both the host immune system and antimicrobial agents. The ability of *H. pylori* to form biofilms within the gastric mucosa is a critical factor in its persistence and resistance to treatment [49]. Studies have shown that DnaK and other chaperone proteins enhance biofilm formation by stabilizing proteins involved in extracellular matrix production [87,88]. This protects the bacteria from direct antibiotic attack and contributes to the chronic nature of *H. pylori* infections, making eradication more challenging.

Moreover, *H. pylori* can acquire antibiotic resistance through horizontal gene transfer [89]. The role of HSPs in this process is still under investigation. However, it is possible to hypothesize that some HSPs—with or without the abetment of extracellular vesicles—may facilitate the transfer of genetic material between bacteria, allowing for the dissemination of resistance traits within bacterial communities. This horizontal gene transfer could lead to the rapid spread of resistance in populations of *H. pylori*, complicating treatment strategies.

Finally, HSPs may also indirectly contribute to antibiotic resistance by supporting the survival of bacterial subpopulations with reduced metabolic activity, commonly referred to as “persister cells” [90]. These cells exhibit tolerance to antibiotics, despite not carrying resistance genes. HSPs thus may help in maintaining the structural integrity of these dormant cells, which can later repopulate the infection site once antibiotic treatment is completed, leading to the recurrence of infection. Furthermore, we can also postulate that the involvement of HSPs in regulating stress responses enables *H. pylori* to adapt to fluctuations in antibiotic concentrations, creating a dynamic resistance mechanism that challenges conventional antibiotic therapy. However, further studies are needed to disprove this hypothesis.

## 8. Clinical Implications and Future Perspectives

**Figure 1:**
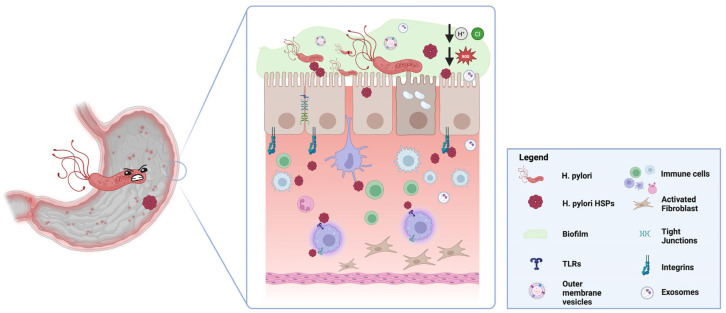
HSPs support bacterial survival under gastric stress by facilitating protein folding, promoting adhesion to gastric epithelial cells, modulating host immune responses, and contributing to antibiotic resistance through efflux pump stabilization and biofilm formation. The muco-microbiotic layer, sticking the epithelial cells of the gastric mucosa, hosts not only bacteria (e.g., *H. Pylori*) but also nanovesicles—produced by both bacteria (OMV) and human cells (exosomes)—which may participate in establishing and perpetuate inflammation. The extracellular vesicles may also carry HSPs (not shown), either from bacterial or host derivation, giving them further chance to have a role in *H. Pylori* infection and complication pathogenesis. Further details are explained in the text.

A schematic representation of *H. pylori* stress response mechanisms mediated by HSPs is summarised in Figure 1. Given their critical role in *H. pylori*’s survival and pathogenesis, HSPs may represent promising targets for novel therapeutic strategies to treat infections caused by this pathogen. These proteins are highly conserved across bacterial species, increasing their potential as drug development targets. Potential approaches to targeting HSPs include using HSP inhibitors or small-molecule inhibitors that specifically block bacterial chaperone activity. Such inhibitors could disrupt the bacterial protein folding machinery, impairing the ability of *H. pylori* to survive under stress conditions and thereby increasing the bacterium’s susceptibility to existing antibiotics.

Developing HSP inhibitors offers several advantages. First, HSPs are involved in various essential bacterial processes, making them attractive candidates for broad-spectrum antimicrobial therapies. By targeting these proteins, it may be possible to weaken *H. pylori* defences across a range of stressors, including acidic environments, oxidative stress, and the presence of antimicrobial agents. Furthermore, because HSPs play a role in forming and maintaining biofilms, inhibiting these proteins could disrupt biofilm integrity, allowing antibiotics to penetrate deeper into bacterial communities and effectively clear infections. HSPs have also been explored as potential vaccine candidates due to their immunogenic properties [91]. Vaccination against *H. pylori* HSPs may enhance immune clearance and reduce infection rates, particularly in regions with a high prevalence of *H. pylori*-associated diseases. Vaccine development targeting GroEL and DnaK, for example, could prime the host immune system to recognize and neutralize *H. pylori* before it establishes a chronic infection. The potential for HSP-based vaccines extends beyond *H. pylori*, as these proteins are also present in other bacterial pathogens, making them ideal candidates for developing universal vaccines. However, molecular mimicry phenomena must be considered while designing these vaccines and appropriate preliminary experimentation is mandatory before using it in trials.

Targeting HSPs in combination with conventional antibiotics could offer synergistic effects, improving treatment efficacy and overcoming antibiotic resistance. A multi-pronged approach that combines antibiotics with HSP inhibitors may help reduce the bacterial load more effectively, prevent the emergence of resistant strains, and promote a more rapid eradication of *H. pylori*. In addition, such combination therapies could be particularly beneficial in refractory or chronic infections, where conventional treatment options often fail. Notably, the involvement of HSPs in vesicle-mediated communication between *H. pylori* and host epithelial cells through OMV and exosomes unveils a novel layer of host–pathogen interaction, offering promising targets for innovative therapeutic interventions.

An emerging research field is the putative use of natural compounds, such as plant-derived molecules or peptides, which putatively can also inhibit *H. pylori* HSP activity during infections. These compounds might offer a more sustainable and cost-effective alternative to traditional synthetic antibiotics and HSP inhibitors. For example, flavonoids and polyphenols have shown promise in disrupting bacterial HSP function, and their use as adjunctive treatments could open new avenues for therapeutical experimentation [92].

## 9. Conclusions

HSPs, including those vehiculated by extracellular vesicles, play a multifaceted and indispensable role in the survival and pathogenicity of *H. pylori*. Their involvement in adhesion, stress resistance, immune modulation, and antibiotic resistance underscores their importance as potential therapeutic targets. Given their central role in bacterial physiology, HSPs are not only critical for the immediate survival of *H. pylori* under hostile environmental conditions but also contribute to its ability to persist and cause chronic infections in the human stomach.

Among the bacterial HSPs, as emerged in this work, GroEL and DnaK are likely the key proteins regulating the majority of *H. pylori*’s functions. Both proteins exhibit notable acid resistance, strong substrate binding affinity, and play crucial roles in maintaining protein homeostasis and interacting with the host. These characteristics are essential for *H. pylori*’s ability to adapt, persist, and cause infection in the gastric environment.

Understanding how HSPs can contribute to *H. pylori*-induced disease pathogenesis is essential for developing novel treatment strategies, particularly in the face of increasing antibiotic resistance. By targeting HSPs with specific inhibitors, vaccines, or combination therapies, it may be possible to improve treatment outcomes and reduce the global burden of *H. pylori*-related diseases, including gastric cancer, a major public health concern worldwide. Furthermore, the study of HSPs in *H. pylori* could provide valuable insights into the broader field of bacterial pathogenesis, paving the way for new therapeutic approaches that target other microbial pathogens as well.

Additionally, the evolving understanding of HSPs’ role in immune modulation and bacterial persistence opens up exciting prospects for developing novel immune-based therapies. By leveraging the immunogenic properties of these proteins, therapeutic strategies that not only eliminate the pathogen but also promote long-term immunity against reinfection might be possible. As research continues to unravel the complexities of HSPs in *H. pylori* and other pathogens, the potential for discovering new antimicrobial agents and vaccine candidates grows, offering hope for more effective treatments in the near future.

## Figures and Tables

**Table 3:** This table summarises the key functions of HSPs in *H. pylori* infection, including their roles in adhesion, colonization, stress resistance, immune modulation, and bacterial persistence. The table outlines the specific HSPs involved, their mechanisms of action, and their effects on bacterial survival and pathogenicity. References correspond to the relevant sections in the main text.

Role in *H. pylori* Pathogenesis	Involved HSP(s)	Putative Mechanism of Action	Putative Effects	References
** Acid stress resistance **	GroEL, DnaK	Maintains protein stability, interacts with urease	Neutralizes gastric acidity, promotes survival	[54]
** Adaptation to temperature variations **	HSPs (various)	Maintains protein homeostasis	Ensures bacterial survival under environmental stress	[59]
** Adhesion and colonization **	GroEL	Interaction with host cell integrins	Facilitates bacterial adhesion and colonization	[46]
** Biofilm formation **	GroEL, other chaperonins	Protein stabilization, regulation of biofilm-related genes	Enhances persistence and antibiotic resistance	[47,48,51,52,53]
** Immune evasion **	HSPs (various)	Apoptosis modulation, macrophage inhibition	Promotes bacterial persistence	[65]
** Immune system modulation **	GroEL	Activation of TLR2/TLR4	Induces chronic inflammation	[60,61]
** Molecular mimicry **	GroEL, GroES	Structural similarity with human HSPs	May trigger autoimmune responses	[62,63,64,65]
** Oxidative stress resistance **	HSPs (various)	Protein stabilization, enhances antioxidant enzyme activity	Protects against ROS, increases bacterial survival	[55,56,57,58]
